# Genetic parameter estimation for reproductive traits in QingYu pigs and comparison of carcass and meat quality traits to Berkshire×QingYu crossbred pigs

**DOI:** 10.5713/ajas.19.0105

**Published:** 2019-10-21

**Authors:** Jia Luo, Yiting Yang, Kun Liao, Bin Liu, Ying Chen, Linyuan Shen, Lei Chen, An’an Jiang, Yihui Liu, Qiang Li, Jinyong Wang, Xuewei Li, Shunhua Zhang, Li Zhu

**Affiliations:** 1 College of Animal Science and Technology, Sichuan Agricultural University, Chengdu, Sichuan 611130, China; 2 College of Animal Science and Technology, Southwest University, Chongqing 400715, China; 3 Bashan Animal Husbandry Technology Co., LTD, Tongjiang, Sichuan 636700, China; 4 Sichuan Province General Station of Animal Husbandry, Chengdu, Sichuang 610066, China; 5 Chongqing Academy of Animal Science, Rongchang 402460, China

**Keywords:** QingYu Pig, Reproductive, Genetic Parameter, Carcass and Meat Quality, Crossbred

## Abstract

**Objective:**

The QingYu pig is well known for its excellent meat quality attributes in Sichuan province, China. In order to improve its production efficiency, the determination of genetic factors contributing to quantifiable economic traits of livestock is important. Moreover, the cross-breeding of QingYu pigs with western breeds possessing strong growth attributes is an efficient way to improve the performance of this breed.

**Methods:**

Here, the genetic parameters of several important reproductive traits of QingYu pigs were estimated, include total number born (TNB), number born alive, litter birth weight, individual birth weight, number of piglets weaned, litter weaning weight, and individual weaning weight. The data was analyzed using the ASReml 3.0 software (NSW Inc., Sydney, Australia). Furthermore, the effects of crossing Berkshire with QingYu (BQ) pigs on carcass and meat quality traits, as well as the effects of slaughter weight on carcass and meat quality of BQ were characterized.

**Results:**

QingYu pigs exhibited superior reproductive traits. The TNB available to QingYu pigs was more than 8 per parity. The observed repeatability of the reproductive traits of the QingYu pigs was between 0.10 and 0.23. The significantly correlated genetic and phenotypic of reproduction traits were consistent. Interestingly, the BQ pigs exhibited improved carcass quality, with a significant increase in loin muscle area, lean percentage and reduction in sebum percentage. As a result, BQ had higher L_45min_, lower cooking scores, and lower drip loss. In addition, the loin muscle area, body length, and sebum percentage were significantly higher in 90 and 100 kg animals. Cooking loss showed a significant increase at 80 kg, and marbling increased significantly from 90 kg.

**Conclusion:**

The results of this study suggest that QingYu pigs exhibit excellent reproductive properties and heritability of these traits. Crossing with Berkshire is an efficient strategy to improve the carcass and meat quality of QingYu pigs for commercial operations. Furthermore, it appears as though the optimal slaughter weight of BQ pigs is at approximately 90 kg.

## INTRODUCTION

The QingYu pig is a typical Southwest Chinese indigenous pig breed, and is primarily distributed throughout the Daba Mountain area of Sichuan Province. This breed is famous for its excellent meat quality attributes. QingYu pigs are a medium-sized breed, and the body is covered with black hair. Furthermore, this breed is classified as a meat and fat dual-purpose breed [1]. However, the preserved population of pure QingYu pig is very small, even it is difficult to find in the main producing areas. QingYu pig is near extinction in China, we only have one protection farm in Sichuan province, and the population is very limited. The breed has fallen into obscurity, near extinction in fact, due to its poor carcass quality, growth attributes, a lack of optimum feeding and management strategies, and the threat posed by the relative lack of popularity compared to modern western commercial breeds does not make the breed attractive in commercial production ventures. At the present time, lots of the breed characteristics of QingYu pigs are still not well understood.

In current commercial breeding programmes, great em phasis is placed on improving reproduction traits. In general, the breeding goal is to increase the number of piglets weaned per sow per year [2]. Lots of reports have shown the effectiveness of selective breeding on litter size [3–5]. In addition to litter size, many other traits affecting reproductive performance could be used to improve a breeding program. In the present study, 7 important reproductive traits were analyzed. The aim was to characterize a comprehensive set of genetic parameters contributing to QingYu pig reproductive function traits for direct use in its future breeding programs.

Moreover, one major objective of swine industry is to increase the lean-to-fat ratio of carcasses [6]. Crossbreeding has been widely used to improve lean growth while maintaining pork quality [7]. QingYu pig is a traditional obese type breed, near extinction in fact, due to its poor carcass quality (the lean percent is about 40%), growth attributes (270 days to 90 kg body weight), a lack of optimum feeding and management strategies. Moreover, Chinese pay more attention to meat quality. To meet the Chinese market requirement, the most common sire line breeds used in China for cross-breeding with native breeds are Duroc and Berkshire. Compared to QingYu pig, Berkshire was used as a terminal sire breed to produce fattening pigs with excellent meat quality, which are desirable for its excellent growth rate and high intramuscular fat content. However, the related information is nonexistent for crosses with QingYu pigs. Therefore, genetic parameters (heritability, genetic correlation and repeatability) of several important reproductive traits of QingYu pigs were characterized, as were the effects of cross-breeding between QingYu and Berkshire pigs on carcass and meat quality traits. Finally, the effects of slaughter weight on carcass and meat quality of Berkshire×Qingyu crossbred (BQ) pigs were assessed to determine its optimal slaughter weight.

## MATERIALS AND METHODS

### Experimental design and animal management

All animal procedures were conducted in accordance with institutional guidelines for the care and use of laboratory animals was approved by the Animal Care and Ethics Committee of Sichuan Agricultural University, Sichuan, China, under Permit number DKY-B20161705.

The experiment was coordinated by Sichuan Agricultural University and conducted in the QingYu pig preservation farm at the Bashan Animal Husbandry Technology Co., LTD. To protect this traditional Chinese Southwestern type indigenous pig breed, we need to know the genetic parameters of this breed. To assess the genetic parameter of reproductive traits, records of 218 QingYu sows (from 38 sires) (the population include all the individuals with complete information we can find) from four generations of a closed QingYu pig protection farm were used in this study. The reproductive performance included all of parities from 1st to 8th in the farm, the average parity was 4.4. All the records were collected between 2011 and 2015 in the QingYu Pig Protection farm and the following data were collected: total number born (TNB), number born alive (NBA), litter birth weight (LBW), individual birth weight (PBW), number of piglets weaned (WN), litter weaning weight (LWW), and individual weaning weight (PWW). The sows were housed in individual pens (2 m^2^) within a larger room. All pigs were fed identical diets twice daily, and were allowed *ad libitum* access to water. The experimental diets, based on corn and soybean meal, were formulated with crude protein concentrations, trace minerals, and vitamins to meet or exceed the National Research Council (NRC, 1998) recommendations for the different growth phases. During the experimental period, the corn–soybean meal diet was offered to pigs as shown in Table 1.

A total of 36 male (barrow) pigs (6 QingYu, 30 BQ) were sacrificed in order to analyze the carcass and meat quality (2 randomly selected animals per litter). More specifically, 6 QingYu and 6 BQ were sacrificed at 210 d, whereas a total of 24 BQ were sacrificed upon reaching 70, 80, 90, or 100 kg (6 pigs per weight) to evaluate carcass and meat properties. The day before slaughter, the animals were weighed and transported to the abattoir. To minimize the stress, the animals were not mixed with unfamiliar pigs during transportation and in lairage. In addition, they were showered with water, and water was available for drinking *ad libitum* during the 12 h lairage prior to slaughter. The pigs were sacrificed by exsanguination after electrical stunning (90 V, 10 s, 50 Hz). Following exsanguination, the animals were dehaired and eviscerated.

### Estimation of heritability and genetic correlation

Litter size was analyzed as the number of TNB and the NBA. Additionally, LBW, WN, and WW were analyzed. The WN was calculated as the percentage of piglets weaned divided by the total number of piglets nursed, including those animals that were cross-fostered. The data was analyzed using the ASReml 3.0 software (NSW Inc., Sydney, Australia) to determine effects of year, parity and season on the 7 described reproduction traits; insignificant parity was combined. Next, the likelihood ratio test was used to analyze the influence of stochastic effects on the reproductive traits. Finally, a mixed animal model was used to estimate the variance of components for each reproductive trait. The variance components of each reproductive trait, corresponding heritability and genetic correlation were estimated using ASReml3.0.

Mixed linear model of reproductive characters in QingYu pigs used was as follows:

$${\text{Y}}_{\text{ijklmn}}=\mu +{\text{Sire}}_{\text{i}}+{\text{Dam}}_{\text{j}}+{\text{Year}}_{\text{k}}+{\text{Parity}}_{\text{l}}+{\text{Season}}_{\text{m}}+{\text{e}}_{\text{ijklmn}}$$

of which, Y_ijklmn_ = character observation value; μ = population mean; Sire_i_ = Boar random effect; Dam_j_ = sow random effect,Year_k_ = year fixed effect; Parity_l_ = parity fixed effect; Season_m_ = season fixed effect; e_ijklmn_ = random residual effect.

The computational formula of heritability (h^2^) and genetic correlation (r_g(xy)_):

$${\text{h}}^{2}=\frac{\sigma_{\text{A}}^{2}}{\sigma_{\text{P}}^{2}}$$

$\sigma_{\text{A}}^{2}$ = additive genetic variance component,

$\sigma_{\text{P}}^{2}$ = variance component of total phenotype.

The significance of genetic force was tested using a t-test, where, $\text{t}=\frac{{\text{h}}^{2}}{\sigma_{{\text{h}}^{2}}}$, σ_h_^2^ = standard error of heredity.

$$\text{rg}(\text{xy})=\frac{{\text{COV}}_{\text{XY}}}{\sqrt{\sigma_{\text{X}}^{2}\sigma_{\text{Y}}^{2}}}$$

COV_XY_, the covariance components of the 2 traits; r(xy) represent the phenotypic correlation, rg(xy) represent the genetic correlation between trait x and trait y. $\sigma_{\text{X}}^{2},\sigma_{\text{Y}}^{2}$ represents two variance components of characters respectively.

Repeatability estimation was determined using the following equation [8]:

$${\text{r}}_{\text{e}}=\frac{{\text{MS}}_{\text{B}}-{\text{MS}}_{\text{W}}}{\left[{\text{MS}}_{\text{B}}+(\text{k}-1){\text{MS}}_{\text{W}}\right]}$$

Where, r_e_ = repeatability;

$\sigma_{\text{B}}^{2}$ = variance components between groups;

$\sigma_{\text{W}}^{2}$ = The components of the within variance; MS_B_ = mean squares between groups; MS_W_ = mean squares within group; k = number of individual measures.

Due to different numbers of observations, the individual measure should be calculated as the weighted average number of k_o_:

$${\text{k}}_{\text{o}}=\frac{1}{\text{D}-1}\times \left(\text{N}-\frac{\Sigma \;{\text{k}}_{\text{i}}^{2}}{\text{N}}\right)$$

where k_i_ = the number of individual measurements; D = the number of individuals; N = the sum of individual measures.

Then, repeatability estimation formula was employed: 

$${\text{r}}_{\text{e}}=\frac{{\text{MS}}_{\text{B}}-{\text{MS}}_{\text{w}}}{\left[{\text{MS}}_{\text{B}}+({\text{k}}_{\text{o}}-1){\text{MS}}_{\text{W}}\right]}$$

The significance of repeatability force was tested by t-test, t = r_e_/σ_re_, σ_re_ represents the standard error of repeatability.

### Carcass measurements

After evisceration, the left half carcass was weighed and the carcass percentage calculated. On the right half carcass, morphometric parameters carcass length was measured using a flexible tape. The loin eye area was measured at the level of the last rib. The backfat thickness was measured with a flexible tape at the level of the first rib, the last rib, and at the point of the backfat thickness, and the mean of these three measurements was used as the backfat thickness value. The sebum percentage was calculated by sebum weight/carcass weight.

### Meat-quality measurement

The meat quality was determined by reference to the previous methods used in our laboratory [9]. Muscle pH, meat color, drip loss, cooking loss and Warner-Bratzler shear force (WBS), were measured 45 min post-mortem and 24 h post-mortem. Marbling scores were evaluated 24 h post-mortem using a published visual standard (NPPC, 1991; USA). Muscle pH was measured at approximately 1 cm below the cutting surface of longissimus dorsi (LD) (3th to 4th rib) using a pH-star meter (SFK Inc., Berlin, Germany). The electrode was calibrated with pH 4.6 and 7.0 buffers equilibrated at 35°C for the measurements of the warm carcass after 45 min and equilibrated at 4°C for the measurements at 24 h. Meat lightness (CIE L*) was also objectively measured in LD cutting surface between the 5th and 6th rib, by using the Model CR-300 Minolta Chroma Meter (Minolta, Ramsey, NJ, USA) fitted with a 50-mm-diameter orifice, using a D65 illuminant, and standardized against a white tile. Drip loss was determined by weighing sliced meat stored at 4°C by using the plastic-bag method after 24 h and calculated as a percentage of the sliced meat original weight. To determine cooking loss, 2.5 cm thick (approximately 100 g) sliced loin samples was cooked to an internal temperature of 70°C in a steamer. Cooking loss was determined by weight difference between uncooked and cooked samples. The WBS was determined using a Texture Analyzer (TA.XT. Plus, Stable Micro Systems, Godalming, UK) equipped with a WarnerBratzler shearing device detailed in our previous studies [6,9]. For determination of intramuscular fat content, 50-g loin meat samples were taken and the intermuscular fat was analyzed using the Soxhlet method.

### Statistical analysis

For the statistical analysis of the results of carcass and meat quality, a general linear model procedure was used (SPSS 22.0, Chicago, IL, USA). When the crossbred effect was significant (p<0.05), Duncan’s test was used at the 5% level to make pairwise comparison among sample means. As no significant interaction was observed between crossbred and slaughter weight for any of the meat traits studied, only main effects are presented. The individual animal was used as the experimental unit for all the data. Values are expressed as means± standard error.

## RESULTS AND DISCUSSION

### Reproduction traits of parity

**Figure 1:**
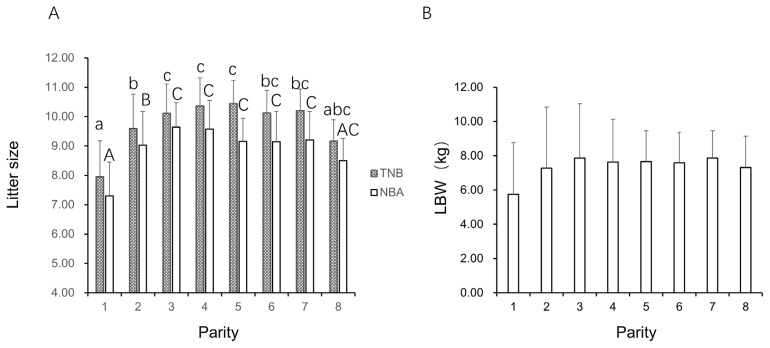
Comparison of reproductive characteristics of QingYu pigs in different parity. Different lowercase letters a, b, and c indicate that TNB is significantly different between parity (p<0.05), while the same letter indicates no significant difference; The capital letters A, B, and C showed significant differences in NBA between parity (p<0.05), while the same letter indicates no significant differences.

As can be seen in Figure 1A, The TNB and NBA at the 1st and 2nd parity of QingYu pigs were lowest, with the 4th and 5th parity were significantly higher than was observed for the 3rd parity (p<0.05). These observed change tendency among parities are consistent with those of other pig breeds [10]. The TNB and NBA at the 3rd to 8th parity exhibited a stable and high level. The TNB peaked during the 4th and 5th parity, reaching 10.37 and 10.44, respectively. The NBA peaked at the 3rd and 4th parity, reaching 9.64 and 9.58, respectively. The TNB and NBA remained a high level at the 7th parity, and began to decline at the 8th parity, although the decrease was not statistically significant. These data suggest that the QingYu pigs did not reach the descending inflection point of litter traits by the 8th litter. As Shen et al [11] reported, parity and breed had significant effects on TNB and LBW. In the present study, the data indicated that TNB available to QingYu pigs was more than 8 parity, and the utilization age of sows was significantly higher than Western pig breeds. These observations are consistent with the previous studies demonstrating excellent reproductive performance of several Chinese indigenous pig breeds [12,13]. As is presented in Figure 1B, the QingYu LBW increased significantly for the 2nd parity, whereas the parameter remained steady for parities 2 to 8. Compared with the significant decrease at the 6th parity for Western pig breeds, the utilization embryos numbers of QingYu pigs were much higher than those in the 6th parity [14,15].

### Heritability and repeatability of reproductive traits

The observed heritability of reproduction traits of QingYu pigs was between 0.09 and 0.22 (Table 2). The heritability of TNB, NBA, BW, and WN were 0.22, 0.11, 0.22, 0.21, respectively, which resulted in a categorization of these parameters as being of low heritability. The t-test of TNB, NBA, BW, PBW, WN heritability indicated significant results. However, WW and PWW did not achieve statistical significance. The heritability of the reproduction traits of QingYu pigs was observed higher than that of other breeds native to China and other western breeds. For example, the heritability of TNB (0.22) was medium, whereas the same trait for other Chinese native breeds was less than 0.2 (low heritability), such as the case for Diannan miniature pigs (0.15) and Huainan pigs (0.12 to 0.2). Chen et al. reported that the genetic parameters and trends for litter traits in US Yorkshire, Duroc, Hampshire, and Landrace pigs exhibited heritabilities for NBA of 0.10, 0.08, 0.09, and 0.08, BW was 0.08, 0.07, 0.08, and 0.09, WN was 0.05, 0.07, 0.05, and 0.05 in the 4 breeds, respectively [16]. The heritability of the TNB, NBA, and WN of the Canadian Yorkshire and Landrace pigs were 0.10 to 0.15, 0.9 to 0.14, 0.06 to 0.08, respectively [17]. The repeatability of reproduction traits of QingYu pigs ranged from 0.10 and 0.23. The above results showed that the heritability observed for a specific breed varied under different environmental conditions, and the heritability of traits between breeds were somewhat variable as well.

With respect to the repeatability of these reproductive traits, that of TNB and BW were the highest (0.23), and the observed repetition of WW was the lowest (0.10). The repeatability of TNB, NBA, BW, and PBW were statistically significant (p< 0.05), although WN, WW, and PWW were not. The repetition of BW, WW, and WN of NanHe pigs were 0.39, 0.32, and 0.34, all higher than QingYu pigs [18]. The repeatability of QingYu pigs was slightly lower than Western pig breeds, and the observed repeatability of the traits were similar to that of other Chinese native breeds [19]. The results of this study showed that the repeatability of the 7 reproductive traits of QingYu pigs exhibited low repeatability, consistent with the regularity of native pig breeds reported in the early domestic studies.

### Genetic and phenotypic correlation of reproductive traits

The genetic correlation between traits is of great significance to the production process. Some important economic traits are difficult to measure *in vivo* (e.g. meat yield, lean meat yield, etc.) and some economic traits are expressed late in development. Furthermore, some of these traits exhibit low heritability (e.g., reproductive traits such as litter number, birth weight, etc.), but are capable of being measured indirectly through their correlation with other traits. As is presented in Table 3, the genetic correlation between reproductive traits was statistically significant. A negative correlation was observed between both TNB and NBA with PBW (−0.43, −0.34, respectively) (p<0.05). The genetic correlation between TNB, NBA, WN, and PBW was −0.69±0.24, −0.17±0.06, and −0.33 ±0.15, respectively. Correlations between the other traits were positive. The genetic correlation between TNB and NBA was 0.72±0.11 (p<0.01). The genetic correlation between WN and TNB, NBA, PBW was 0.81±0.38, 0.87±0.23, and 0.28± 0.14, respectively (p<0.05). Finally, TNB exhibited a significant positive correlation between NBA, WN, WW in the context of phenotypic expression (p<0.001). Previously, Roehe and Kennedy [17] estimated the genetic parameters of Canadian Yorkshire and Landrace pigs using a bivariate animal model. This model inferred that the genetic correlation of Yorkshire between TNB and NBA was 0.99, NBA and WN was 0.72, for Landrace between TNB and NBA was 0.95, and between NBA and WN was 0.66. The genetic correlation between reproductive traits of QingYu pigs was consistent with these results, indicating that the higher TNB and NBA were associated with a reduction of BW. When BW increased, the WN, WW, and PWW were increased. Moreover, the significantly correlated genetic and phenotypic of traits that were consistent between TNB vs NBA, WN vs NBA, WW vs TNB, WN vs NBA, WW vs WN, and PBW vs NBA.

### Comparison of carcass traits between QingYu and Berkshire×QingYu cross pigs

The carcass traits selected for comparison between the different breeds at 270 d of age are presented in Table 4. There were significant differences in dressing percentage (p<0.05) between breeds. Although the values are lower than that of commercial breeds (Pietrain [79.6%], Landrace Belga [80.1%], Large White [78.6%]) [20], and in other native pig breeds, such as Chato Murciano (77.7%) [21]. The highest values were observed in BQ (70.79%). The highest lean percentages were observed in the crossbreeds, whereas the sebum percentages of the breeds were lower (p<0.05). For morphometric parameters, the carcass length and backfat thickness were not significantly different between QingYu and BQ pigs (p>0.05). In addition, loin muscle area was significantly higher and backfat thickness was significant lower in BQ (p<0.001). The measurements of sebum indicated a decline of fattening in QingYu crossbreeds. Kim et al [22] reported that the primary differences between indigenous and modern pig breeds at the same age are carcass weight and loin cross-sectional area. Ruusunen [23] found that there is great heterogeneity in the traits studied in each breed and crossbreed. Therefore, in the present study, each trait was analyzed separately. Here, our findings between pure QingYu and crossbred corroborate with several other studies.

### Comparison of meat quality traits between QingYu and Berkshire×QingYu pigs

The effect of crossbreeding on meat quality traits of QingYu pigs are presented in Table 5. The data indicated no significant differences in pH values between pure QingYu pigs and crosses in the *longissimus dorsi* muscle, which is consistent with other previous reports [24]. Loins from BQ crossbred pigs had higher L45 min, with lower drip loss and cooking loss (p<0.05). The pH value and meat color of BQ pigs were both within the range of high quality pork, and no pale soft exudative or dark firm dry meat were found. Among the breeds examined, the water holding capacity (WHC) of crossbred BQ was significantly higher than Qingyu pigs. The WHC affects the eating quality, particularly when considering juiciness. Furthermore, high WHC values could be advantageous to the pork processing industry for processed meats and in the perceived appearance of fresh meat to the consumer [25]. Therefore, the higher WHC of the meats from BQ indicate that the meat was of excellent quality.

### Comparison of carcass traits of Berkshire×QingYu pigs at different slaughter weights

**Figure 2:**
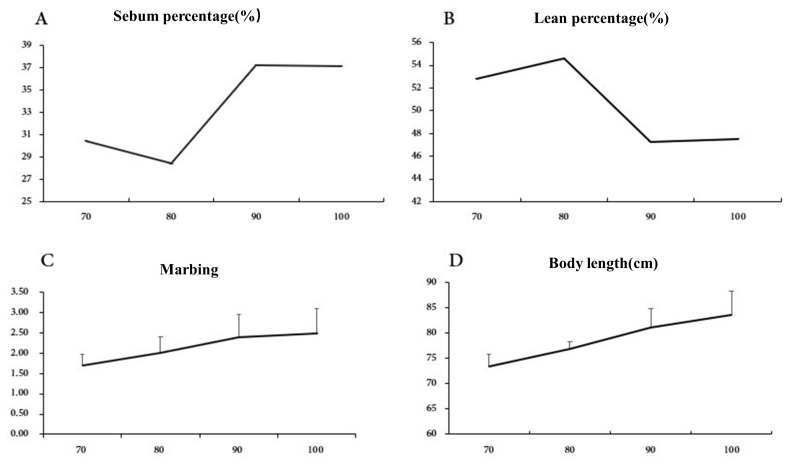
The law of carcass traits and meat quality traits in different body weight. (A) Sebum percentage (%); (B) Lean percentage (%); (C) Marbing; (D) Body length (cm).

To determine the optimal slaughter weight of BQ pigs to send to market, the effects of slaughter weight on carcass traits, morphology measurements and primal cuts are presented in Table 6. The backfat thickness and sebum percentage both increased with weight. There were similar dressing percentages observed in BQ in four slaughter weight (p>0.05). Both Cisneros et al [26] and Weatherup et al [27] noted there was no significant effect of slaughter weight on carcass weight or dressing percentage. The loin muscle area, body length, sebum percentage increased significantly for animal weights of 90 and 100 kg, whereas the lean percentage was significantly decreased (p<0.05). The increase in loin muscle area with weight supports previous reports. These observations are similar to previous reports in which the lean meat percentage decreased with increasing slaughter weight [28]. However, loin muscle area, body length, sebum percentage and lean percentage were not significantly different between the 90 and 100 kg body weights (p>0.05). These results showed that before the weight of 90 kg, BQ pigs were in a phase of rapid muscle growth. Conversely, fat deposition was slow. As a result, BQ pigs had a high percentage of lean mass coupled with rapid muscle growth. As can be seen in Figure 2A and 2B, the variation of lean percentage and sebum percentage at different slaughter weight stages, the 90 kg weight was an inflection point. Therefore, slaughter before the inflection point of 90 kg is conducive to taking advantage of the superior performance of high lean percentage of BQ pigs.

### Comparison of meat quality traits of Berkshire×QingYu pigs at different slaughter weights

As is presented in Table 7, a comparative analysis of the meat quality of BQ pigs at different slaughter weights revealed differences in cooking loss and marbling. Compared with meat from 70 kg animals, the cooking loss showed a significant increase at 80 kg. In contrast, the differences between 80, 90, and 100 kg body weights were not significant (p>0.05). Marbling increased significantly at the 90 kg body weight. The data reported here are in agreement with other studies showing that as the slaughter weight increases, some aspects of meat quality increase [29]. Therefore, based on the comprehensive indicators, the meat quality of BQ pig was best at a slaughter weight of 90 kg.

## CONCLUSION

It can be concluded from this study that the utilization embryo numbers of QingYu pig were more than 6th parity. In QingYu pigs, the significantly correlated genetic and phenotypic of reproduction traits were consistent. Crossbreds between Berkshire and QingYu pigs showed improvements in reproduction traits and provided a higher meat lean mass percentage. Although purebred QingYu pigs possessed good meat quality, crossbreeding with Berkshires improved the meat quality traits. The meat quality of BQ pig was best at a slaughter weight of 90 kg.

## Figures and Tables

**Table 1:** Ingredients and nutrients of the basal experiment diets

Items	20 to 50 kg	50 to 120 kg
Ingredients (g/kg)		
Corn	700	772
Soybean	270	200
CaHPO_4_	9	8
Limestone	8	7
Salt	3	3
Lysine	0.3	-
Premix1)	10	10
Nutrients		
DE (MJ/kg)	13.9	13.83
CP (g/kg)	180	154
Ca (g/kg)	6	5.2
P (g/kg)	5	4.6
Lysine (g/kg)	9.5	7.5

DE, digestible energy; CP, crude protein.

1) Provided the following (unit/kg): 1,600 mg of Cu, 10,000 mg of Fe, 3,000 mg of Mn, 10,000 mg of Zn, 40 mg of I and 30 mg of Se, 605,000 IU of vitamin A, 155,000 IU of vitamin D_3_, 1,800 IU of vitamin E, 200 mg of vitamin K_3_, 300 mg of vitamin B_1_, 400 mg of riboflavin, 200 mg of vitamin B_6_, 1.5 mg of vitamin B_12_, 1,500 mg of pantothenic acid, 2,800 mg of niacin and 12,500 mg of choline.

All data were analyzed values except DE, which was calculated using swine National Research Council (1998) values.

**Table 2:** The heritability and repeatability of reproductive on Qingyu pig

Traits	n	X̄	s	h^2^±SE	r_e_±SE
TNB	1,166	9.53	2.26	0.22±0.06*	0.23±0.05*
NBA	1,154	8.83	2.18	0.11±0.05*	0.14±0.04*
BW	1,154	7.22	1.93	0.22±0.06*	0.23±0.05*
PBW	1,154	0.81	0.12	0.18±0.06*	0.20±0.05*
WN	937	8.18	1.63	0.21±0.09*	0.22±0.12
WW	937	59.87	11.51	0.09±0.12	0.10±0.09
PWW	937	6.86	0.92	0.18±0.11	0.19±0.12

SE, standard error; TNB, total number born; NBA, number born alive; LBW, litter birth weight; PBW, individual birth weight; NW, weaning piglet, LWW, weight of weaning litter; PWW, weaning weight.

* Represent significant level at 0.05 (p<0.05).

**Table 3:** Genetic and phenotypic correlations between reproductive traits in Qingyu pig

Items	TNB	NBA	PBW	WN	WW	PWW
TNB	-	0.72±0.11**	−0.43±0.10*	0.81±0.38*	0.79±0.09**	−0.69±0.24
NBA	0.89±0.01**	-	−0.34±0.06**	0.87±0.23*	0.48±0.08**	−0.17±0.06*
PBW	0.16±0.06*	−0.26±0.06*	-	0.28±0.14*	0.67±0.18*	0.87±0.21*
WN	0.68±0.05**	0.25±0.02**	−0.08±0.09	-	0.89±0.15**	−0.33±0.15*
WW	0.57±0.06**	0.03±0.003**	−0.03±0.09	0.74±0.04**	-	0.04±0.01
PWW	−0.12±0.09	−0.13±0.05*	0.12±0.09	−0.32±0.08*	0.39±0.08*	-

The above diagonals in the table are the genetic correlations between traits, while the below diagonals are the phenotypic correlations between traits.

TNB, total number born; NBA, number born alive; LBW, litter birth weight; PBW, individual birth weight; NW, weaning piglet; LWW, weight of weaning litter; PWW, weaning weight.

* Represents significant level (p<0.05), and

** represents extremely significant level (p<0.01).

**Table 4:** Comparison of carcass traits between Qingyu pig with BQ pig

Items	Qingyu	BQ
n	6	15
Age (d)	210	210
Weight (kg)	67.58±6.12^a^	80.11±5.37^b^
Backfat thickness (cm)	2.75±0.21^a^	2.88±0.58^a^
Loin muscle area (cm^2^)	18.35±1.88^a^	23.25±4.38^b^
Body length (cm)	78.00±1.41^a^	75.78±2.38^a^
Dressing percentage (%)	63.31±0.76^a^	70.79±2.10^b^
Lean percentage (%)	50.29±6.23^a^	53.32±3.86^b^
Bone percentage (%)	10.94±1.09^a^	11.38±1.47^a^
Sebum percentage (%)	37.58±7.62^a^	30.59±4.71^b^

BQ, Berkshire×Qingyu crossbred pigs.

a,b The letters in the table are marked with the same letter indicating no significant difference in carcass characteristics at different weight stages, while different letters indicating significant.

**Table 5:** Comparison of meat quality traits between Qingyu pig with BQ

Items	Qingyu	BQ
n	6	15
Age (d)	210	210
Weight (kg)	67.58±6.12^a^	80.11±5.37^b^
pH_45min_	6.47±0.21^a^	6.72±0.19^a^
pH_24h_	5.98±0.23^a^	5.55±0.08^a^
L_45min_	38.03±1.52^a^	42.34±1.70^b^
L_24h_	42.33±1.82^a^	46.15±4.37^a^
Drip loss (%)	4.56±0.26^a^	2.03±.051^b^
Cooking loss (%)	66.59±3.11^a^	65.23±3.21^b^
Marbing	3.52±0.32^a^	2.17±0.5^a^

BQ, Berkshire×Qingyu crossbred pigs.

a,b The letters in the table are marked with the same letter indicating no significant difference in carcass characteristics at different weight stages, while different letters indicating significant.

**Table 6:** Carcass traits of Berkshire×Qingyu pig crossbred in different body weight

Items	Weight stage			

	70	80	90	100
Weight (kg)	70.2±1.64	81±1.30	92.6±1.52	101.6±2.60
Backfat thickness (cm)	2.52±0.64^a^	2.63±0.30^a^	2.83±0.52^a^	3.03±0.60^a^
Loin muscle area (cm^2^)	20.23±5.05^a^	24.07±4.91^a^	26.82±6.52^b^	29.75±7.66^b^
Body length (cm)	73.4±2.51^a^	76.8±1.48^a^	81.1±3.75^b^	83.7±4.60^b^
Dressing percentage (%)	70.32±0.02^a^	71.45±0.02^a^	71.72±0.03^a^	70.37±0.01^a^
Lean percentage (%)	52.84±0.02^a^	54.56±0.03^a^	47.27±0.01^b^	47.53±001^b^
Sebum percentage (%)	30.45±0.02^a^	28.41±0.01^a^	37.24±0.02^b^	37.18±0.02^b^

a,b The letters in the table are marked with the same letter indicating no significant difference in carcass characteristics at different weight stages, while different letters indicating significant.

**Table 7:** Meat quality traits of Berkshire×Qingyu pig crossbred in different body weight

Items	Weight stage			

	70	80	90	100
pH_45min_	6.68±0.34^a^	6.69±0.24^a^	6.63±0.20^a^	6.45±0.33^a^
pH_24h_	5.52±0.12^a^	5.54±0.09^a^	5.60±0.10^a^	5.65±0.06^a^
L_45min_	44.27±2.06^a^	43.15±0.93^a^	43.32±2.56^a^	43.57±2.40^a^
L_24h_	45.69±1.65^a^	48.10±4.30^a^	47.39±4.40^a^	49.19±3.67^a^
Drip loss (%)	0.03±0.01^a^	0.02±0.01^a^	0.02±0.01^a^	0.02±0.01^a^
Cooking loss (%)	0.62±0.03^a^	0.65±0.01^b^	0.64±0.02^b^	0.64±0.02^b^
Marbing	1.7±0.27^a^	2.00±0.41^a^	2.40±0.55^b^	2.50±0.61^b^

a,b The letters in the table are marked with the same letter indicating no significant difference in carcass characteristics at different weight stages, while different letters indicating significant.
